# Medical Opinion and Movement

**Published:** 1909-11-20

**Authors:** 


### Medical Opinion and Movement.

At a meeting of the Berlin Society of Medicine, Dr. Blumberg drew attention to a special sign as a means of Differential Diagnosis between Appendicitis and Perityphilitis. This sign consists in the pain evinced on suddenly withdrawing pressure over the appendicular region of the abdomen. The hand is pressed slowly but firmly upon the abdomen over McBurney's point and then suddenly withdrawn It has been shown by Lennander that while the peritoneum in a normal condition is insensitive, when inflamed it is extremely sensitive to any dragging movement, such as would be produced in the manner described above. This sign would therefore be diagnostic of a peritoneal inflammation. Of course, other signs of appendicits must be considered in conjunction, for, as the author points out, the sign may be present in affections of the uterine appendages. But, according to the author, this sign will only be present in a greater or less degree according to the involvement of the peritoneum in the inflammatory process. The pain produced by withdrawal of the hand, or what is called "decompression," must, of course, be compared with that resulting from "compression." In some cases of peritonitis compression and decompression may both produce about the same amount of pain over a certain area of the abdomen, except at one point, where decompression pain is much more marked, and this would indicate the subjacent viscus as the prime focus of the inflammation.

In a paper contributed to the *Medical Record*, Dr. Leonard Ely makes some interesting observations based upon a clinical and pathological study of forty-five specimens of Resected Joints. Of ten cases in which tuberculosis was not present, nine were clinically diagnosed as such, it is stated by men of considerable ability. On the other hand, four joints proved to be tuberculous on pathological examination, although the clinical diagnosis was otherwise. These observations arouse a sceptical view in the author's mind in regard to reported statistics of tuberculous joints cured by conservative methods. Another interesting fact is the comparatively large proportion of primary synovial tuberculosis—four out of fifteen cases in which the origin of the process could be traced. Microscopic examination showed the efforts of nature against the disease by depriving the joint of motion and converting it into a synostosis, and Dr. Ely concludes from this that the best way to cure a tuberculous joint is by immobilisation, and he deprecates the efforts of some surgeons to preserve motion in the treatment of these cases. He advocates the plaster of Paris bandage rather than traction splints. In case of operation he thinks it unnecessary to "go wide of the disease" as advocated by some surgeons. If the joint be firmly immobilised, small foci of disease will generally heal spontaneously. On the other hand, the author is very emphatic upon the utter inutility of the curetting operation as usually practised.

The latest therapeutic purpose for which Adrenalin has been utilised is in the treatment of severe vomiting of pregnancy. Freund has applied it locally to the nasal mucous membrane on the ground that he has found a hyperæmic condition of this part in a large proportion of pregnant women, and he argued that vaso-motor disturbances caused by the condition of pregnancy might lead to an upset of the vomiting centre owing to its proximity in the medulla. Dr. T. Silvester, of Modena, however, administers the adrenalin internally. He based his justification for the treatment upon the fact that severe and persistent vomiting often occurs in cases of Addison's disease, and upon certain experiments he has conducted upon pregnant rabbits and guinea-pigs, in which a suprarenal capsule was removed. In the case reported, that of a woman in whom the vomiting was so persistent and uncontrollable that all known forms of treatment entirely failed, administration of the drug brought about complete cessation of the vomiting in two days. Similar successful cases are reported by Dr. Zanfrognini, also of Modena, and also by Dr. S. Rebaudi. The latter's case was that of a young woman who at the third month of pregnancy was in such an exhausted condition by the persistent and uncontrollable vomiting that abortion had been definitely decided upon. Adrenalin was, however, first tried. Ten drops of a 1 in 1,000 adrenalin solution were given twice a day The effect was marked, and at the end of a fortnight the dose was reduced to a half. This was continued for another ten days, when the condition of the patient became quite normal, and she was delivered at term of a well-developed child.

In recent years the Test Meal has been losing a good deal of the importance at one time attached to it in gastric diagnosis, and there has been a growing distrust in its findings for the differential diagnosis of gastric cancer and gastric ulcer. This loss of confidence would appear to be justified by the results of observations carried out by Drs. Christopher Graham and Donald Guthrie and reported in the *New York Medical Journal*. These observations extend over 250 cases of ulcer of the stomach and duodeuum and 150 cases of carcinoma. In all these examination by the test meal was carried out, and all of them were eventually operated upon and the correctness of the diagnosis was established. Seventy-five per cent. of the patients operated upon for gastric ulcer did not show high gastric acidity. This was especially true of older patients with chronic ulcer. The younger subjects with developing ulcers usually showed high acid percentages. Of the 150 cases of gastric cancer free hydrochloric acid was absent in only 80, lactic acid was present in 64, and food remnants in 63. Among the conclusions drawn from these observations is the fact that while free hydrochloric acid is rarely present in advanced carcinoma, in the earlier stages the test meal may show little or no change from the normal. Decided food remnants are of greater significance as a surgical indication than the acid percentages. Like all other clinical signs, the test meal has only a relative value, as the authors point out, and is only useful in conjunction with the rest of the clinical picture; but to postpone operation till the test meal gives a definite pathological result would often mean to delay till it is too late.

---

DIABETES has been considered by some people to be an infectious disease of intestinal origin, so Dr. Feletti, of Catania, sets forth, in the *Policlinico*, his investigations into the matter. In the course of these he repeated the experiments of Topfer, devised to combat the conclusions arrived at by von Noorden. Dogs and rabbits were fed by the mouth with small quantities of the fæces of diabetics much diluted with sterile bouillon. The mixture was kept for several days in an incubating chamber before being administered to the animals. After a certain time these animals showed a glycosuria, which lasted a few days. Control animals fed on normal fæces and bouillon prepared in the same way, showed no traces of glycosuria. Subsequently the author endeavoured to find out whether it is possible to discover in the blood, fæces, and urine of diabetics a micro-organism, capable of cultivation, which will produce glycosuria in animals. After many unsuccessful attempts he was finally able to isolate from the urine of a particularly grave case of diabetes a micro-organism which he was able to grow on bouillon, and which produced a glycosuria lasting some days when administered to dogs or rabbits, either by the mouth or intravenously. A second administration of the culture to animals who had recovered from the first, led to a second appearance of glycosuria. The cultural and morphological characters of the micro-organism somewhat resembled those of the streptococcus pyogenes aureus of Roosing. Feletti does not claim to have discovered the specific agent of glycosuria, but insists on the importance of the foregoing facts.

---

THE work of H. Roger on the Saccharification of Inuline by the human saliva would seem to prove that the fermentation of this isomeride of starch is due, not to the effects of a salivary ferment, but to those of an organism present in the mouth. This observer has, in fact, discovered a streptococcus, consisting of chains of diplococci. These chains are often long, and will not develop at the temperature of the surrounding air nor in solid media. The activity of their growth is markedly influenced by the presence of sugar in the medium, and is directly proportional to the amount of sugar added. They will grow in water, to which a solution of levulose, glucose, saccharose, or lactose, has been added. Milk is not a favourable medium, but a fairly abundant vegetation can be produced from blood-serum, the medium remaining alkaline. The vitality of the organism is very variable, but it is rapidly killed if the medium becomes acid. This streptococcus has no reducing power on starch, does not seem pathogenic, and is only interesting because of its fermentative action.

THE site of election for the performance of the operation of Paracentesis Abdominis (after ascertaining by percussion that there is no intestinal resonance in the neighbourhood) is usually taught to be the mid-point between the umbilicus and the anterior superior iliac spine. Jeunet, in *La Province Médicale*, questions the suitability of the choice of this point. Against it he advances the objections that paracentesis performed at this point is painful owing to the numerous nerve filaments present in the neighbourhood, and, moreover, that the trochar is quite likely to encounter an important vessel, either in the parietes, or in some deeper-lying structure, if there be but little fluid present in the abdomen. The same objections hold good when the point selected lies at the junction of the outer with the middle third of a line joining the points mentioned above. The point Jeunet would advise to be taken lies midway between the umbilicus and the top of the symphysis pubis. Here the only structure likely to be injured is the bladder, an accident which can easily be obviated by emptying that viscus beforehand with a catheter. No important arteries or nerves are present at this point, and, although it has been objected that the intestines may be easily injured there, the same objection would seem to hold whatever point be selected, and careful percussion should largely obviate such accidents. Although it is more difficult to evacuate all the fluid by this route, this fact scarce can rank as a serious objection, seeing that it is usual to leave a certain amount of fluid behind, partly to prevent hæmorrhage, partly to stop rapid re-accumulation. It is scarcely necessary to mention that a gravid uterus is a contra-indication to median puncture.

---

EMERICH has recently published in the *Münch. Med. Woch.* an account of his theory that the intoxication of cholera is due to the nitric acid set free in the system by the action of the cholera vibrio in changing the nitrates of the food into nitrites. The starting point of this theory is the fact, pointed out by Petri, that the cholera vibrio has this power, and the author believes that foods such as cucumbers, radishes, cabbages, turnips, etc., which are rich in nitrates, are acted upon in the body in this way by the bacillus of cholera. Moreover, these conclusions are equally applicable to cases of cholera nostras and of infantile cholera, for the bacteria of both diseases have this acid-forming power. It is true that the bacillus coli has the same action, but as it normally only inhabits the large intestine, where nitrates rarely penetrate, it usually is unable to determine any nitric intoxication. Cases will, however, be remembered where for purposes of radiography massive doses of subnitrate of bismuth have been given with fatal results. These latter are explicable by supposing that the drug has reached the large intestine unchanged, where contact with the bacillus coli has set up a fatal acid intoxication. The author has been able experimentally to reproduce in dogs and guinea-pigs the symptoms of cholera, by dividing them into three groups and introducing into the stomachs of the first, second and third groups respectively doses of cholera bacilli, nitrates, and a mixture of bacilli and nitrates. The animals of the first two groups showed no morbid symptoms, whereas those of the third group showed characteristic nitrate intoxication, as evidenced by diarrhœa, cyanosis, convulsions, hypothermia and the presence of nitrates in the blood on spectroscopic and chemical examination. The author has been able to supplement these experiments by personal observation at St. Petersburg of a recent epidemic of cholera. He has been able to prove the presence in considerable quantity of this acid in the aqueous vomit which patients throw up at the onset of the disease. In the algid period the reaction is feeble or altogether wanting. The dejecta of cholera patients usually contain little or no acid since this has been decomposed already by the leucin tyrosin and albumins of the intestine. Treatment therefore consists, in the first place, in helping the elimination of the acid formed by washing out the stomach with alkaline solutions or one containing meta-diamido-benzol, which forms non-toxic compounds with nitric acid. Prophylaxis, however, plays the chief part in the struggle against the disease, and the most useful prophylactic measure after securing as far as possible a pure water supply, consists in preventing the ingestion of food rich in nitrates.

---

Puncture of the Lung as a diagnostic measure is warmly advocated in the *Lancet* by Dr. T. J. Horder. The indications are described as three in number: when careful examination of the sputum in a case of consolidation of the lung fails to reveal the nature of the disease; when abscess is diagnosed; and in bronchiectasis. In both the latter conditions the sputal flora are so varied that the casual micro-organism is difficult of detection. The technique is practically the same as that of diagnostic puncture of the pleural cavity, and the contents of the syringe are to be both cultured and also submitted to immediate microscopical examination in films. Thus may be obtained information of great service in cases of abscess of the lung, pneumonia (especially following septic processes elsewhere, such as appendicitis, perforated gastric ulcer and the like), broncho-pneumonia, and acute pulmonary tuberculosis, in which sputum is so frequently absent. At a time, says the author, when vaccine therapy appears to be so promising in dealing with many localised infective conditions, the prompt isolation of the causal organism becomes of paramount importance. When the affected organ is the lung, the investigation of its condition by direct puncture may be the sole means of obtaining the necessary culture from which to prepare the vaccine.

---

At a recent meeting of the Medical Society of the District of Columbia, Buckner Randolph gave a statistical analysis of 157 cases of Phthisis. Stress was laid upon the comparative indifference of the negro both to the early symptoms of the disease and to the measures necessary for its arrest. Bad hygienic conditions, gregarious habits, and indifference are responsible for the high comparative incidence and mortality among negroes, rather than any inherent race susceptibility. He noted a large proportion of elderly patients (207 over 40 years of age), generally those who had had the disease many years. Fifty-six per cent. gave a history of definite house exposure to human infection. If true histories were given the percentage would be even higher. Dr. Randolph deprecated the practice of basing diagnosis on sputum findings, on the ground that patients learned to consider negative findings as proof against the disease, and thus lost their chance of getting well. His results in 43 cases, who made as many as five visits to the dispensary, were in all stages: 4 cured, 8 arrested, 24 improved, and 7 not improved. He claimed that, whatever provision might be made in the way of hospitals, sanatoria, etc., that the bulk of consumptives would remain in their houses until they were too advanced to respond to treatment. He urged that more attention be given to home treatment along the lines of tuberculosis classes inaugurated by Dr. Pratt, of Boston. He drew attention to the importance of early disinfection of foci, and the futility of mere terminal disinfection as generally practised.

---

The Gastric Capacity of Infants is believed by Mosenthal to be very usually underestimated. In the *Archives of Pediatrics* he publishes tables upon which his opinion is based, and gives reasons which account for the actual capacity of a healthy infant's stomach being considerably greater than that deduced from post-mortem measurements. In a series of twenty-four cases of very young marasmic infants treated at the New York Foundling Hospital, he found that the average quantity ingested was rather larger than the average size of the stomachs as given for the respective ages in textbooks; and he points out that these infants, being under-developed and weakly, can hardly be supposed to have stomachs of the same capacity as healthy ones. He says that soon after milk is taken into a baby's stomach the pyloric half—or antrum pylori—elongates in consequence of peristaltic waves from left to right, and is thus enabled to accommodate more food. Also that within five to eight minutes of ingestion small quantities of the stomach contents are forced through the pylorus into the duodenum. Since milk curdles completely within ten minutes of being swallowed, the fluid whey requires no more gastric digestion, and passes on into the intestine, whereas the curds remain in the stomach for two hours at least in the case of mother's milk and three hours when cow's milk is given. The deduction from all these arguments is that small babies should be fed with larger quantities and at longer intervals than those commonly advised. For breast-fed infants the minimum between feeds should be two and a half hours, and three hours is better at quite a tender age; for bottle-fed babies it should be three hours from the first. The quantity of milk for each feed should exceed the measured average gastric capacity for the age of the child by a considerable margin. It is said that gastric dilatation can thus successfully be avoided.

